# A case of TAFRO syndrome with a large mediastinal mass treated with debulking surgery

**DOI:** 10.1186/s40792-016-0188-8

**Published:** 2016-06-17

**Authors:** Masaaki Nagano, Jun Matsumoto

**Affiliations:** Department of Thoracic Surgery, NTT Medical Center Tokyo, 5-9-22 Higashi-Gotanda, Shinagawa-ku, Tokyo, 141-8625 Japan

**Keywords:** Castleman’s disease, TAFRO syndrome, Anterior mediastinal mass, Pleural effusion

## Abstract

Multicentric Castleman’s disease is a polyclonal lymphoproliferative disorder. Recently, a new variant of the disease was reported and named TAFRO syndrome, an acronym for thrombocytopenia, ascites, myelofibrosis, renal dysfunction, and organomegaly. A 55-year-old woman presented to our hospital with dyspnea on exertion and high fever. Laboratory tests revealed anemia, thrombocytopenia, and proteinuria. Computed tomography (CT) revealed a large anterior mediastinal mass, mild splenomegaly, bilateral pleural effusion, pericardial effusion, and mild systemic lymphadenopathy. A CT-guided biopsy was unable to establish a definitive diagnosis, so we resected the mediastinal mass for diagnostic and therapeutic purposes. Pathological findings were consistent with the hyaline vascular type of Castleman’s disease (CD), and she was diagnosed with TAFRO syndrome. There has been no description of a patient with TAFRO syndrome with a large mass, and this is the first case of TAFRO syndrome treated with debulking surgery.

## Background

Castleman’s disease (CD) is a rare, non-neoplastic lymphoproliferative disorder first reported in 1956 [1]. Multicentric Castleman’s disease (MCD) is a CD subtype with multiple lesions and systemic symptoms that includes a heterogeneous group of disorders with various etiologies. A new disease entity—TAFRO syndrome—has been proposed as a rare variant of MCD [2], which poses serious diagnostic and therapeutic challenges because its precise pathophysiology remains unknown.

## Case presentation

**Fig 1:**
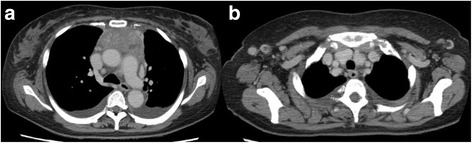
**a** Chest computed tomography demonstrating an anterior mediastinal mass measuring 65 × 35 mm, bilateral pleural effusion, and **b** mild peripheral lymphadenopathy

**Fig 2:**
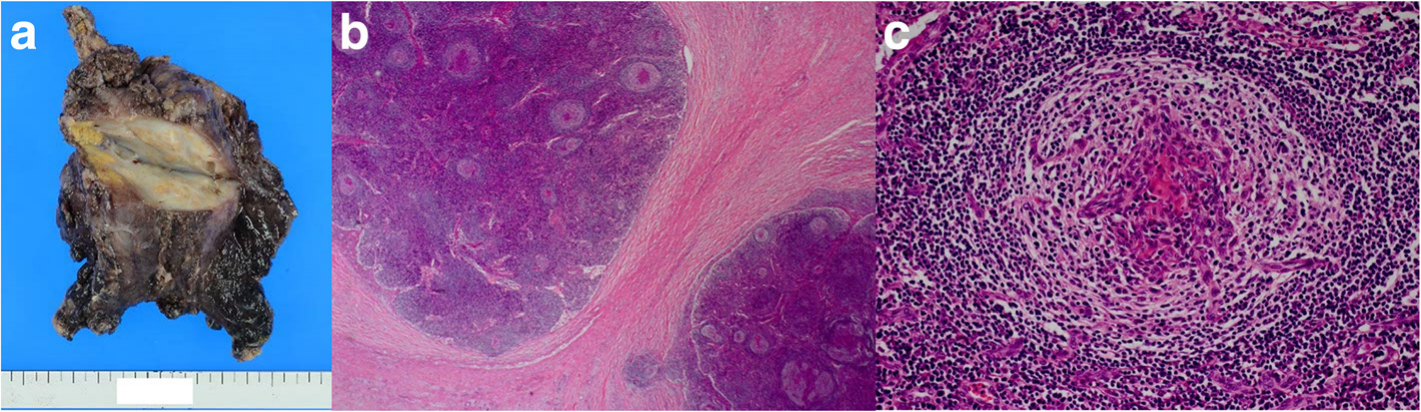
**a** The resected specimen reveals a grayish white tumor that tightly adhered to the left brachiocephalic vein and the pericardium. **b** Pathologic findings of the resected specimen show collagenous fibrous tissue with scattered lymph follicles (hematoxylin and eosin stain, original magnification ×10). **c** The lymphoid follicles were surrounded by a broad mantle zone composed of concentric rings of small lymphocytes and the germinal centers exhibited hyalinized vascular proliferation (hematoxylin and eosin stain, original magnification ×100)

A 55-year-old Japanese woman was referred to our hospital with dyspnea on exertion and high fever for 2 weeks. She had a rapid weight gain from 60 to 80 kg in a month and severe anasarca. Laboratory tests revealed anemia, thrombocytopenia, proteinuria, and elevated levels of serum C-reactive protein, alkaline phosphatase, and interleukin-6. She tested negative for the human immunodeficiency virus (HIV), Epstein–Barr virus, and human herpesvirus-8 (HHV-8) but positive for platelet-associated immunoglobulin G and had a positive direct Coombs test. Computed tomography (CT) revealed a large anterior mediastinal mass, mild splenomegaly, bilateral pleural effusion, pericardial effusion, and mild systemic lymphadenopathy (Fig. 1). Bone marrow examination showed normal cellularity but mild myelofibrosis. A CT-guided biopsy of the mediastinal mass was unable to establish a definitive diagnosis, showing only severe fibrosis with a few spindle cells. Despite treatment with all types of diuretics, including furosemide, tolvaptan, and eplerenone, her medical condition deteriorated. Therefore, we decided to resect the mediastinal mass for diagnostic and therapeutic purposes, considering the possibility of a paraneoplastic syndrome due to the mediastinal mass. A complete resection of the mass via median sternotomy was performed with concomitant resection of the pericardium and left brachiocephalic vein, which were tightly adhered to the mass (Fig. 2a). Histological and immunohistochemical findings revealed that the mass was composed of collagenous fibrous tissue with scattered lymph nodes, and hyperplasia of lymphoid follicles was seen in the lymph nodes (Fig. 2b). The lymphoid follicles were surrounded by a broad mantle zone composed of concentric rings of small lymphocytes and the germinal centers exhibited hyalinized vascular proliferation (Fig. 2c). These pathological findings were consistent with the hyaline vascular type of CD.

**Fig 3:**
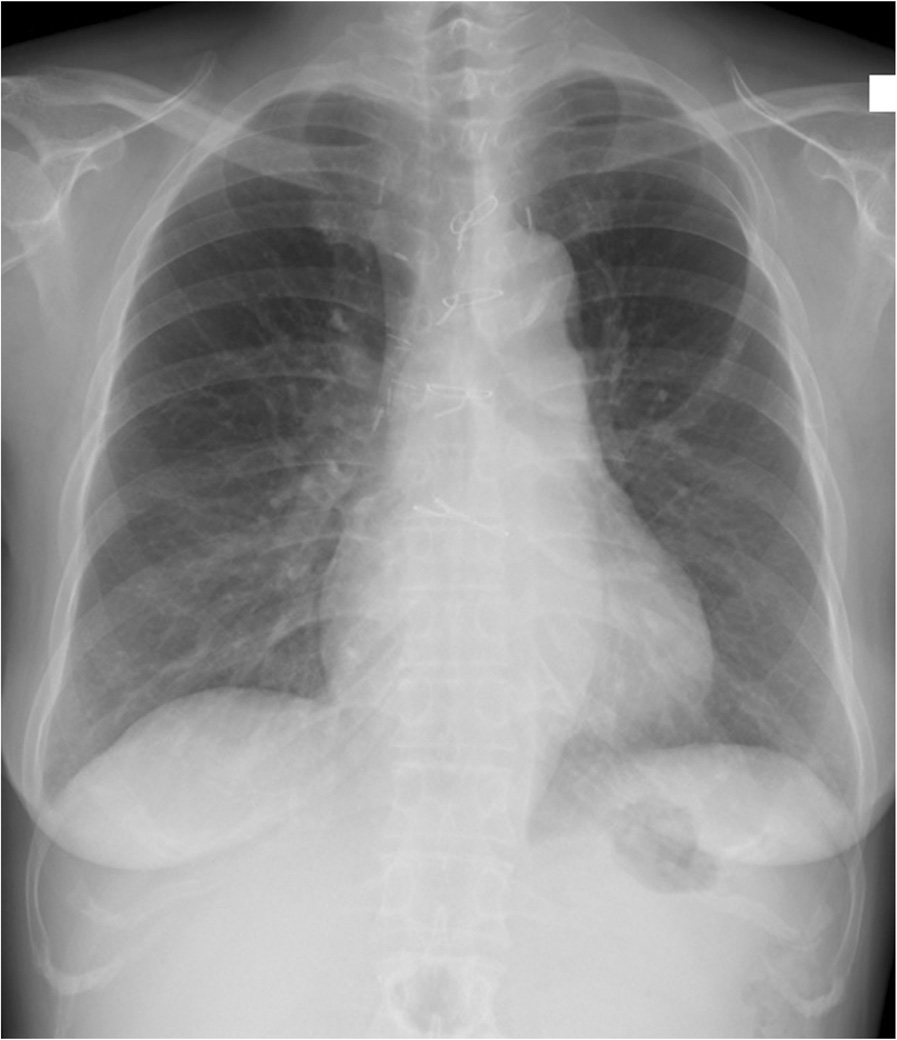
Chest roentgenogram obtained 3 months after surgery

**Fig 4:**
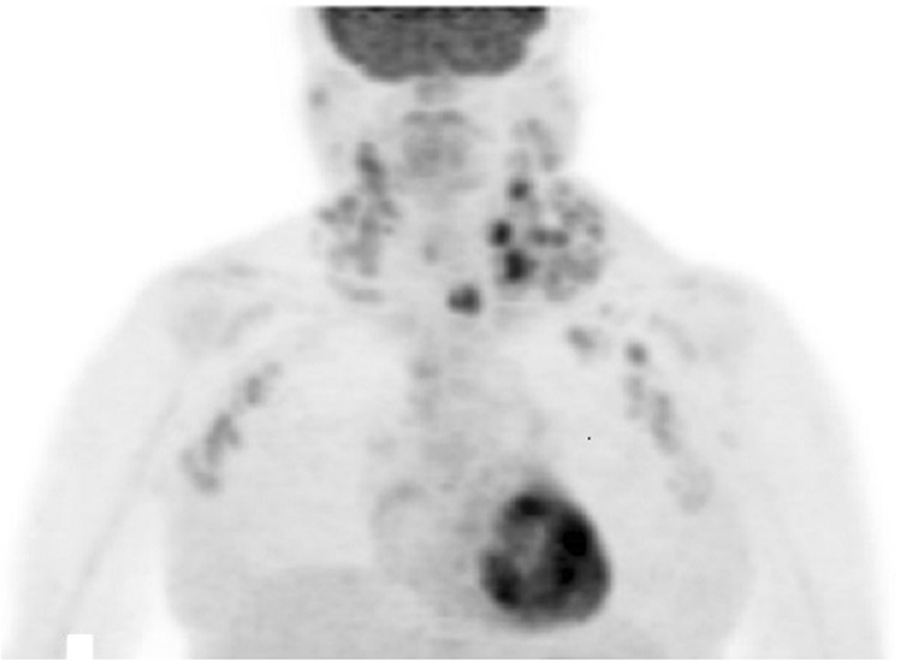
FDG-PET/CT imaging demonstrated higher FDG uptake in cervical and supraclavicular lymph nodes

She had an uneventful recovery from surgery and required limited diuretic therapy. Three months after surgery, she showed a drastic weight loss from 80 to 55 kg and her anemia and thrombocytopenia had resolved. A chest roentgenogram taken at that time point showed no pleural effusion or cardiac enlargement (Fig. 3). However, half a year after surgery, she had rapid weight gain and bilateral ascites. Laboratory tests showed a sharp decrease in hemoglobin and platelets. ^18^F-fluorodeoxyglucose positron emission tomography (FDG-PET)/CT imaging showed higher FDG uptake in the bilateral cervical and supraclavicular lymph nodes (Fig. 4). Excisional biopsy of the cervical lymph node revealed the histological diagnosis of hyaline vascular type CD. Finally, she was diagnosed with TAFRO syndrome based on the laboratory tests, clinical features, and pathological findings. She was admitted to the hematology department of our hospital and received corticosteroid pulse therapy with methylprednisolone. She recovered smoothly and maintained a stable condition with oral corticosteroids 1 year after surgery.

CD cases are pathologically classified into three types: hyaline vascular, plasma cell, and mixed [3]. Unicentric CD is often asymptomatic and associated with the hyaline vascular histological type. However, MCD often demonstrates episodic systemic inflammatory symptoms and multiple organ impairment due to excessive proinflammatory cytokines, such as interleukin-6 [4]. The diagnosis of MCD is established when histopathological lymph node features of CD and clinical features are observed. Therefore, MCD describes a heterogeneous group of disorders with various etiologies [5].

The HHV-8 and HIV infections are a well-established cause of hypercytokinemia in MCD patients with plasma cell histology [6]. However, a group of HIV-negative and HHV-8 negative MCD cases has been reported [7] and defined as idiopathic MCD [5]. Idiopathic MCD pathogenesis is poorly understood, but polyneuropathy, organomegaly, endocrinopathy, monoclonal gammopathy, and skin changes (POEMS syndrome), paraneoplastic syndromes, and secretion of other cytokines by malignant plasma cells have been reported to co-occur with idiopathic MCD [5].

The histopathological and systemic features in idiopathic MCD are thought to be secondary to hypercytokinemia caused by these diseases, and the new disease concept of TAFRO syndrome was recently proposed in Japan as a unique clinicopathological variant of idiopathic MCD. TAFRO syndrome was described in a group of idiopathic MCD patients with thrombocytopenia, ascites, myelofibrosis, renal dysfunction, and organomegaly [2]. This syndrome also demonstrates milder lymphadenopathy, mixed or hyaline vascular histopathology, normal or mildly elevated levels of interleukin-6, presence of autoantibodies, severe anasarca, and normal immunoglobulin levels. The clinical and pathological features in our case were compatible with TAFRO syndrome, but there has been no description of a patient with TAFRO syndrome with a large mass. This is then the first case of TAFRO syndrome with a large anterior mediastinal mass.

Treatment strategies differ for unicentric CD and MCD. Although surgery is the gold-standard treatment for unicentric CD presenting at any organ, the combination of surgery and medical therapy is often used in MCD patients [8]. Three medical treatment strategies have been used in MCD patients with TAFRO syndrome: anti-inflammatory and immunosuppressive therapies such as corticosteroids and cyclosporine A, cytotoxic elimination of cells responsible for hypercytokinemia with cyclophosphamide and doxorubicin, and blockade of interleukin-6 signaling with monoclonal antibodies such as tocilizumab [5]. Surgery in MCD patients is performed to obtain tissue for a full histopathological diagnosis because sufficient specimens of lymph nodes or solid organs are required for a histopathological analysis of CD. The other role of surgery in MCD patients is for debulking, i.e., to excise the majority of the tissues affected by the disease. No long-term survival difference in MCD patients treated with debulking surgery, immunosuppressive therapy, or a combination of both has been demonstrated, suggesting that debulking surgery could be potentially beneficial for the treatment of MCD [8]. Here, mediastinal tumor resection was effective to improve the patient’s symptoms for 6 months. Although TAFRO syndrome may require systemic therapy in the long term, debulking surgery for patients with TAFRO syndrome with a main tumor is thought to be very useful in terms of both diagnosis and treatment.

## Conclusions

In conclusion, we describe the first case of a patient with TAFRO syndrome with a large anterior mediastinal mass. The surgical approach was very beneficial in terms of diagnosis and treatment because it enabled us to confirm the TAFRO syndrome and transiently control disease progression with a resection of the large mediastinal mass.

## Consent

Consent was obtained from the patient for the publication of this case report.
